# Radiation-Induced Dual Oxidase Upregulation in Rat Heart Tissues: Protective Effect of Melatonin

**DOI:** 10.3390/medicina55070317

**Published:** 2019-06-27

**Authors:** Bagher Farhood, Akbar Aliasgharzadeh, Peyman Amini, Hana Saffar, Elahe Motevaseli, Saeed Rezapoor, Farzad Nouruzi, Dheyauldeen Shabeeb, Ahmed Eleojo Musa, Ghorbangol Ashabi, Mehran Mohseni, Habiballah Moradi, Masoud Najafi

**Affiliations:** 1 Departments of Medical Physics and Radiology, Faculty of Paramedical Sciences, Kashan University of Medical Sciences, 8715988141 Kashan, Iran; 2 Department of Radiology, Faculty of Paramedical, Tehran University of Medical Sciences, 1416753955 Tehran, Iran; 3 Clinical and Anatomical Pathologist at Tehran University of Medical Science, Imam Khomeini Hospital Complex, 1419733141 Tehran, Iran; 4 Department of Molecular Medicine, School of Advanced Technologies in Medicine, Tehran University of Medical Sciences, 1416753955 Tehran, Iran; 5 Department of Medical Radiation Engineering, Science and Research Branch, Islamic Azad University, 1477893855 Tehran, Iran; 6 Department of Physiology, College of Medicine, University of Misan, 62010 Misan, Iraq; 7 Department of Medical Physics, Tehran University of Medical Sciences (International Campus), 1416753955 Tehran, Iran; 8 Department of Physiology, Faculty of Medicine, Tehran University of Medical Sciences, 1416753955 Tehran, Iran; 9 Radiology and Nuclear Medicine Department, School of Paramedical Sciences, Kermanshah University of Medical Sciences, 6715847141 Kermanshah, Iran

**Keywords:** radiation, melatonin, IL-4, *Duox1*, *Duox2*, heart

## Abstract

*Background*: Radiation-induced heart injury can lead to increased risk of heart failure, attack, and ischemia. Some studies proposed IL-4 and IL-13 as two important cytokines that are involved in late effects of ionizing radiation. On the other hand, these cytokines may, through upregulation of *Duox1* and *Duox2*, induce chronic oxidative stress, inflammation, and fibrosis. In this study, we evaluated the upregulation of *Duox1* and *Duox2* pathways in hearts following chest irradiation in rats and then detected possible attenuation of them by melatonin. *Materials and Methods*: Twenty male Wistar rats were divided into four groups: (1) control; (2) melatonin treated (100 mg/kg); (3) radiation (15 Gy gamma rays); (4) melatonin treated before irradiation. All rats were sacrificed after 10 weeks and their heart tissues collected for real-time PCR (RT-PCR), ELISA detection of IL-4 and IL-13, as well as histopathological evaluation of macrophages and lymphocytes infiltration. *Results*: Results showed an upregulation of IL-4, *IL4ra1*, *Duox1*, and *Duox2*. The biggest changes were for *IL4ra1* and *Duox1*. Treatment with melatonin before irradiation could attenuate the upregulation of all genes. Melatonin also caused a reduction in IL-4 as well as reverse infiltration of inflammatory cells. *Conclusion*: *Duox1* and *Duox2* may be involved in the late effects of radiation-induced heart injury. Also, via attenuation of these genes, melatonin can offer protection against the toxic effects of radiation on the heart.

## 1. Introduction

Heart tissue is one of the late responding tissues to ionizing radiation. Cardiovascular diseases are among the common non-cancerous disorders for people who have been exposed to ionizing radiation [1]. The most obvious examples of radiation-induced heart diseases include increased risk of heart failure, attack, and ischemia among Chernobyl, Hiroshima, and Nagasaki survivors [2,3]. Increased risk of cardiovascular diseases has been observed in lung and left breast cancer patients [4]. Some radiobiological studies have reported that damages to other organs such as the respiratory system or kidneys may lead to changes in normal physiological functions of the heart and induce heart damage markers [5]. Studies have reported that pathological heart changes may take months to years after exposure to ionizing radiation to appear. In addition, the incidence of heart injury has a direct relationship with radiation dose [6]. 

Although the exact mechanisms of radiation-induced cardiovascular injury have not been completely defined, reports show that infiltration of inflammatory cells plays a key role in chronic oxidative stress, collagen deposition, and changes in the normal structure of heart tissue. Macrophages and lymphocytes are common inflammatory cells following exposure to ionizing radiation. Emerging evidence has also proposed a key role for mast cells [7]. Inflammatory cells induce the release of several cytokines, such as IL-1, IL-2, IL-4, IL-6, IL-8, IL-13, TNF-α, and TGF-β, which mediate continuous reactive oxygen species (ROS) and nitric oxide (NO) production through reduction/oxidation reactions [8]. However, the regulation of these cytokines and downstream genes may be tissue dependent. Chronic oxidative stress and inflammation lead to fibrosis and hypertrophy, which may disrupt normal heart function and blood supply to the heart muscle [9]. 

Recent studies have proposed that IL-4 and IL-13 through upregulation of *Duox1* and *Duox2* induce chronic oxidative stress and fibrosis [10]. The main receptors for IL-4 and IL-13 are *IL4ra1* and *IL13ra2*. In addition to *Duox1* and *Duox2*, IL-4 induces upregulation of TGF-β, a potent stimulator of pro-oxidant enzymes and fibrosis [11]. In the current study, we aimed to evaluate the upregulation of two pro-oxidant genes, Duox2 and Duox1, following irradiation of the heart tissue of rats. 

A previous study by Hassani et al. showed that upregulation of these pathways, especially a long-term increase in *Duox1*, leads to continuous ROS production and genomic instability in thyroid carcinoma cells [10]. This may indicate that in addition to pathological damage to irradiated organs, the increased level of IL-4 or IL-13 and downstream genes trigger genomic instability and carcinogenesis. Duox1 and Duox2 are two membrane-dependent subfamilies of oxidoreductase enzymes that catalyze conversion of oxygen (O_2_) to hydrogen peroxidase (H_2_O_2_) [12]. These two enzymes are very similar and are able to generate a large amount of H_2_O_2_ following activation [12]. Abnormal upregulation of these enzymes may lead to the disruption of normal functions of cells and also an increase in oxidative injury [13]. 

Some natural or chemical agents have been used to reduce radiation-induced heart injury [14,15]. Melatonin is a natural, low toxic hormone that has shown potent radioprotective effects. One of the main effects of melatonin against radiation-induced toxicity is its potent antioxidant defense. It can directly or indirectly neutralize ROS and NO produced by ionizing radiation and pro-oxidant enzymes. The indirect effect of melatonin is mediated via stimulation of the antioxidant defense, including glutathione (GSH), glutathione peroxidase (GPx), superoxidase dismutase (SOD), glutathione reductase (GR), and catalase (CAT) [16]. Melatonin has also shown the ability to suppress chronic inflammation and oxidative stress following exposure to ionizing radiation [17]. The enhancement of the DNA damage response by melatonin has been suggested to prevent triggering of inflammatory reactions following exposure to radiation [18]. Melatonin can also suppress overexpression of pro-oxidant enzymes such as cyclooxygenase-2 (COX-2), nicotinamide adenine dinucleotide phosphate (NADPH) oxidase, and inducible nitric oxide synthase (iNOS) [19]. These are associated with reduced release of pro-inflammatory and pro-fibrosis cytokines [19]. Interestingly, it has been reported that melatonin may induce different effects such as depletion of GSH and induction of cell death in some cancer cells [20]. The differential effects of melatonin on healthy and cancerous cells make it a potential candidate for use as an adjuvant in radiotherapy [18].

In the present study, melatonin was used to reduce radiation-induced heart injury. In addition, the modulation of IL-4 and IL-13 signaling pathways were also detected.

## 2. Materials and Methods

### 2.1. Experimental Design

Melatonin was obtained (Merck company, Darmstadt, Germany) and dissolved in 20% ethanol. For melatonin administration, 1 cc of this solution (100 mg/kg) was injected to each rat intraperitoneally. In this study, 20 adult Wistar male rats (200 ± 20 g) were purchased from Razi Institute, Tehran University of Medical Sciences, Tehran, Iran. All animals were housed in suitable conditions, which included the following: temperature (23 ± 2 °C), humidity (55%) and the same light/dark cycles (light 08:00 to 20:00 and dark 20:00 to 08:00) to prevent any effect of light/dark on basal levels of melatonin. The rats were randomized into four groups (5 rats in each) as follows: group 1: control; group 2: melatonin treated; group 3: radiation; group 4: melatonin treated + radiation. Melatonin was administered 30 minutes before irradiation. A ^60^Co gamma ray source was used to deliver 15 Gy to the heart. Ten weeks after irradiation, all rats were sacrificed to extract their heart tissues. Ventricles were fixed in 10% formalin while their auricles were frozen for real-time PCR.

### 2.2. Real-Time PCR

Total mRNA was isolated from frozen heart tissues of all groups using a TRIzol reagent (Sinagene, Tehran, Iran) while cDNA templates were synthesized using a cDNA synthesis kit (Geneall, Seoul, South Korea) according to the manufacturer’s instructions. The expressions were performed using Corbett real-time PCR (USA). Primers were designed using Generunner software (Hastings Software. Inc. Hastings, NY, USA) and NCBI BLAST. The forward and reverse sequences of primers are shown in Table 1.

### 2.3. ELISA 

The levels of both cytokines IL-4 and IL-13 were detected using an ELISA kit (Zellbio, Krantorweg, Berlin, Germany) according to manufacturer’s instructions. 

### 2.4. Pathological Study

Fixed heart tissues were embedded in paraffin blocks and then sliced into 5 µm sections. Slides were stained using hematoxylin and eosin (H&E) for evaluating infiltration of macrophages and lymphocytes. Histopathological assessments were carried out with the aid of a light microscope.

### 2.5. Statistical Analysis

All statistical analyses were conducted using SPSS software version 16 (SPSS, Inc, Chicago, IL, USA). The significance of the mean ± standard deviations for ELISA and histopathological evaluations were analyzed using the ANOVA test with post hoc Tukey’s HSD, while real-time PCR results were analyzed using the T-test. The *p* values < 0.05 were considered statistically significant.

### 2.6. Ethical Approval

This study was approved by ethical committee of Kashan University of Medical Sciences, Kashan, Iran, with ethical code IR.KAUMS.NUHEPM.REC.1397.006, Approval Date: 2018-05-07.

## 3. Results

### 3.1. Real-Time PCR

**Figure 1:**
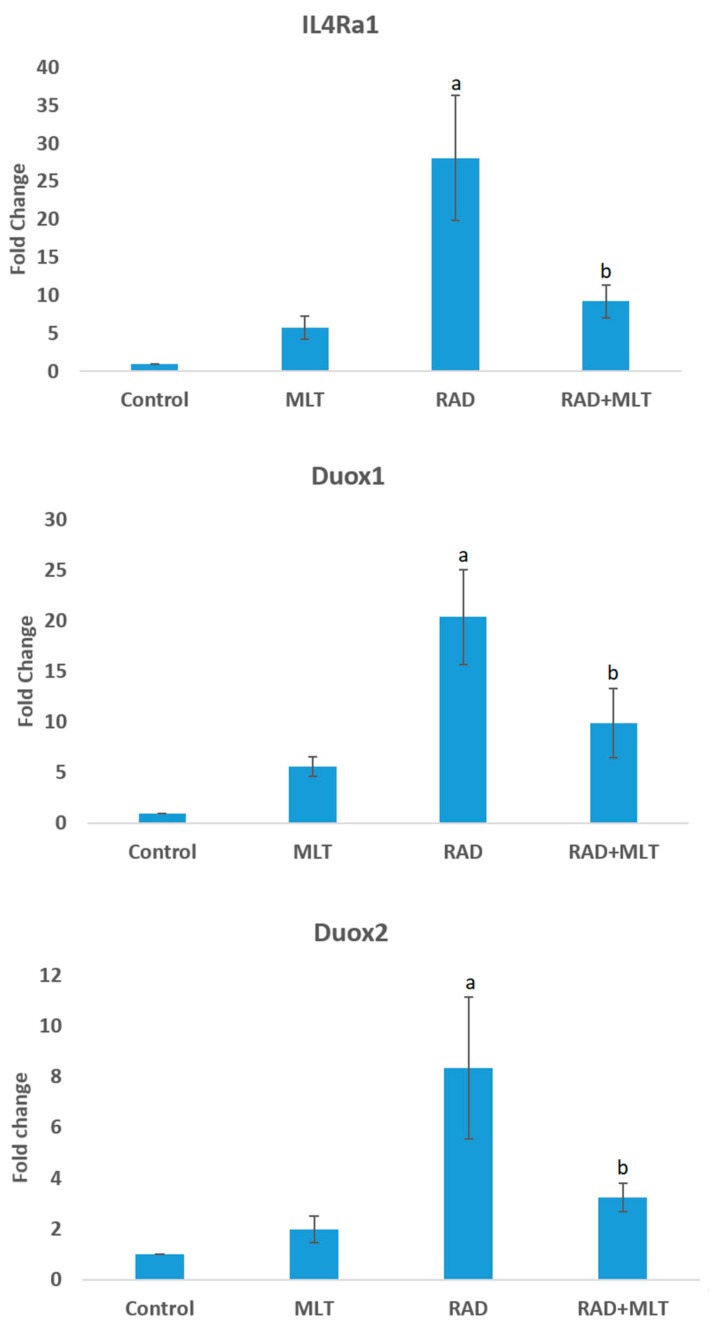
The expression of *IL4ra1*, *Duox1*, and *Duox2* in rats’ heart following melatonin treatment, irradiation, and melatonin treatment before irradiation. The expressions of genes were normalized compared to *PGM1* as an internal control. a: significant compared to control group; b: significant compared to radiation group, (T-test, *p* < 0.05).

Results of the real-time PCR showed a significant increase in the expression of *IL4ra1* compared to the control group (28 ± 8.20) (*p* < 0.05). Melatonin administration before exposure to ionizing radiation led to a significant reduction in *IL4ra1* compared to the control group (9.2 ± 2.15) (*p* < 0.05). Irradiation of heart tissues caused a significant increase in the expression of *Duox1* (20.3 ± 4.6) (*p* < 0.05). When rats were treated with melatonin, the expression of *Duox1* was significantly attenuated (9.8 ± 3.4) (*p* < 0.05). Irradiation of rats’ heart tissues also caused a significant increase in *Duox2* gene expression (8.35 ± 2.8) (*p* < 0.05). Melatonin administration attenuated its expression (3.22 ± 0.55) compared to the radiation group (*p* < 0.05) (Figure 1).

### 3.2. ELISA

**Figure 2:**
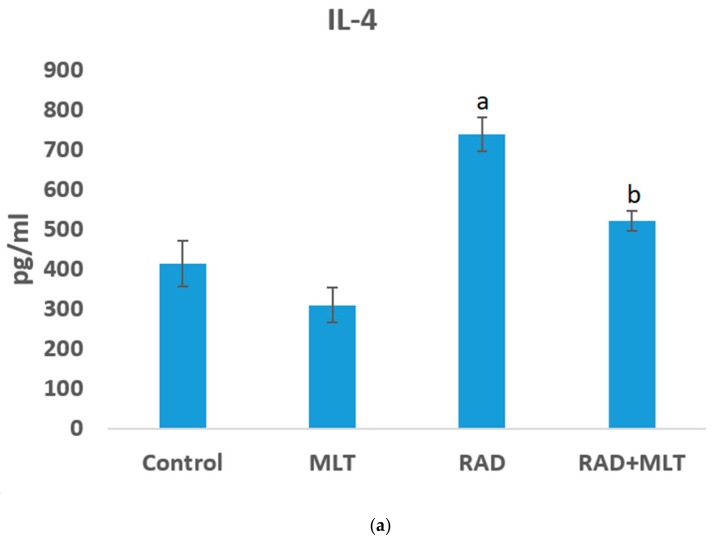

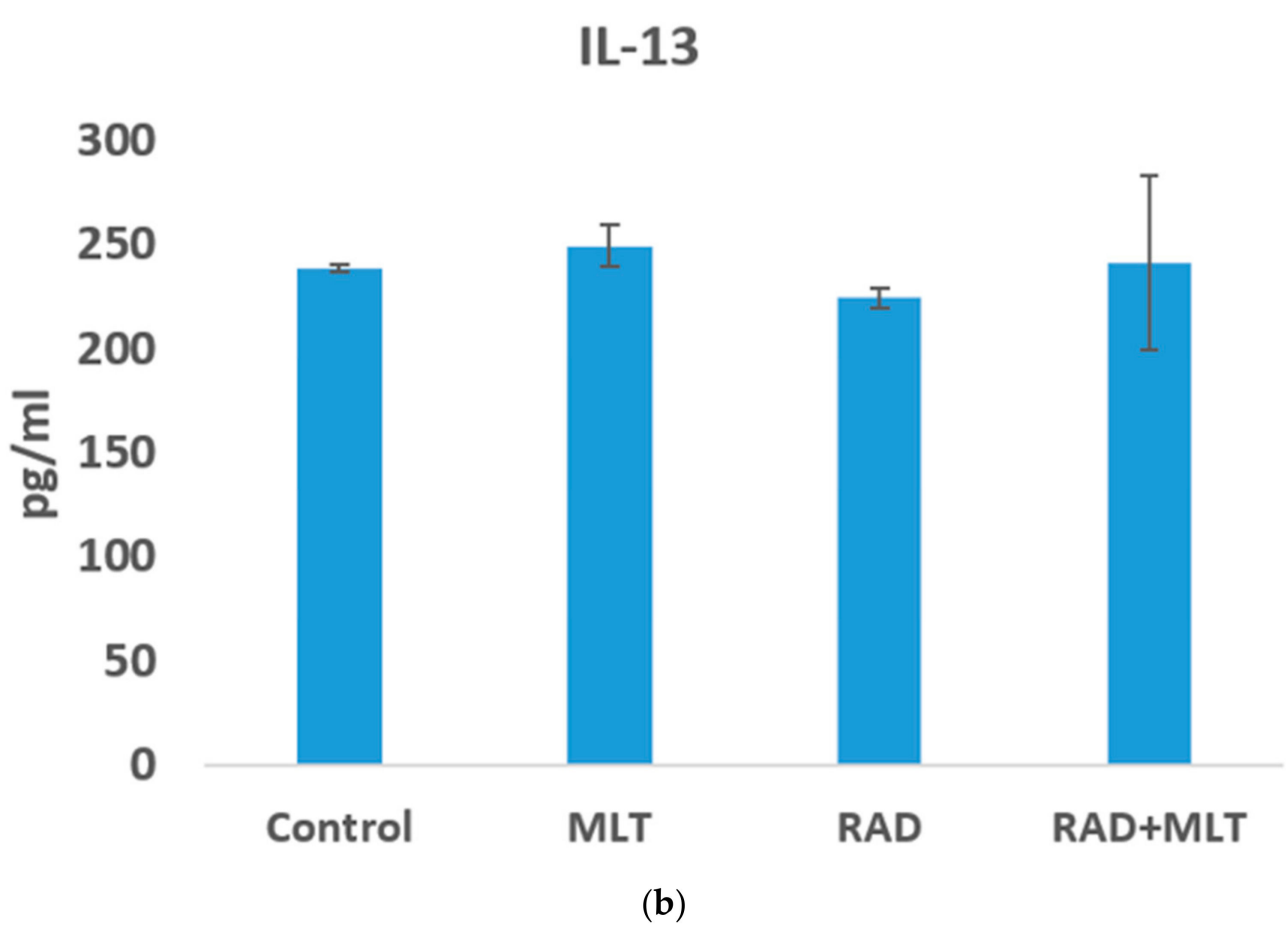
The levels of IL-4 (**a**) and IL-13 (**b**) in control, melatonin (MLT), radiation (RAD), and radiation + melatonin (RAD+MLT) groups. Results show an increase in IL-4 following irradiation of rats’ heart tissues. However, IL-4 decreased when melatonin was administered before irradiation. Results show no significant change in IL-13 for all groups; a: significant compared to control group; b; significant compared to radiation group, (ANOVA, Tukey’s HSD, *p* < 0.05).

IL-4 and IL-13 are low half-life cytokines of 19 min and 1.4 hour, respectively [21,22]. However, they may vary for various species. ELISA assay results showed that irradiation of rats’ heart tissues caused a significant increase in the level of IL-4 (739 ± 42 pg/mL) compared to the control group (414 ± 57 pg/mL) (*p* < 0.01). Treatment with melatonin before irradiation led to a significant decrease in IL-4 contents of cells (522 ± 26 pg/mL) (*p* < 0.01). Treatment with melatonin alone did not induce any significant change. In the case of IL-13, no significant change was observed for any of the groups (Figure 2).

### 3.3. Histopathological Evaluation

**Figure 3:**
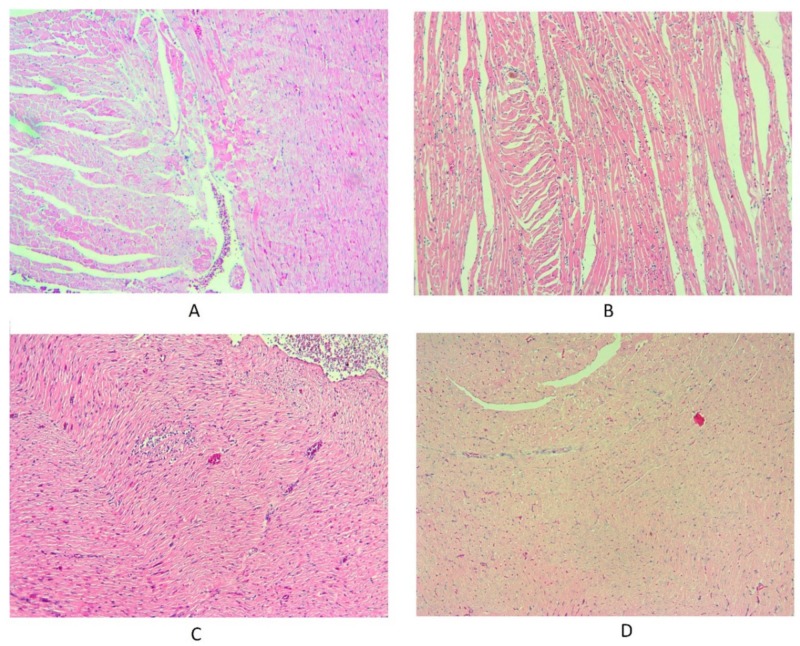
Irradiation of the hearts of rats caused a mild infiltration of inflammatory cells, especially lymphocytes. In the melatonin + radiation group, there was no sign of infiltration of inflammatory cells. (**A**) control; (**B**) melatonin treated; (**C**) radiation; (**D**) melatonin + radiation (hematoxylin and eosin (H&E) staining).

Histopathological study showed a mild infiltration of macrophages and lymphocytes following irradiation of rats’ heart tissues. This was more obvious for lymphocytes. However, when rats were treated with melatonin before irradiation, results showed no infiltration of these cells in heart tissues (Figure 3).

## 4. Discussion

In the current study, we evaluated the upregulation of two important ROS producing agents (*Duox1* and *Duox2*) following heart irradiation. These genes are affected by immune system mediators, especially IL-4 and IL-13. As IL-13 and IL-4 are two important pro-fibrotic cytokines, we hypothesized that chronic upregulation of these cytokines may be associated with increased upregulation of *Duox1* and *Duox2*. It has also been confirmed that IL-4 causes maintenance of macrophages in irradiated tissues. These changes can stimulate redox reactions, leading to chronic oxidative stress, inflammation, and damage to normal heart function. We observed that irradiation of rats’ heart tissues led to increased levels of IL-4 but not IL-13. Real-time PCR showed that irradiation caused significant upregulation of IL4ra1, *Duox1*, and *Duox2*. These changes were obvious for *IL4ra1* and *Duox1*. In addition, histopathological evaluation showed infiltration of lymphocytes and macrophages following exposure to radiation. 

Suppression of inflammatory and pro-fibrotic cytokines have been studied for amelioration of radiation injury in irradiated tissues [23,24]. Inhibition of IL-4 has been shown to alleviate radiation injury, especially infiltration of macrophages in the lung [25]. In addition, inhibition of *Duox1* can attenuate chronic oxidative stress, genomic instability, and late effects of exposure to radiation [10]. In the current study, we used melatonin to attenuate upregulation of *IL4ra1*, *Duox1*, and *Duox2* following irradiation of rats’ heart tissues. Results showed that treatment with melatonin before irradiation reduces upregulation of these genes. Melatonin can also attenuate increased levels of IL-4 and infiltration of lymphocytes and macrophages. 

Melatonin, which was used as a radioprotector in the present study, is an interesting agent for use as a low-toxic adjuvant for patients that undergo radiotherapy. Melatonin can be proposed as an appropriate radioprotector for early and late effects of ionizing radiation in various organs. It is able to attenuate increased level of inflammatory and pro-fibrotic cytokines following exposure to radiation or chemotherapy agents. These effects result from the reduction of upregulation of transcription factors including signal transducer and activator of transcriptions *(STATs)* and nuclear factor-κB *(NF-κB)* [26]. Melatonin is able to blunt translocation of NF-kB into the nucleus, thus preventing upregulation of *COX-2*, *NADPH oxidase* subfamilies (*NOX2* and *NOX4*), and *iNOS* [27]. Via protection of the mitochondria against radiation-induced mitochondrial DNA (mtDNA) injury, melatonin prevents increased inflammasomes and further secretion of IL-1 and IL-18 [28]. Melatonin can attenuate the activity of macrophages [29]. As macrophage activity plays a key role in the release of pro-fibrotic cytokines such as TGF-β, IL-4, IL-10, and IL-13, melatonin may have a beneficial effect in the prevention of the late effects of ionizing radiation through modulation and maintenance of macrophages [30]. Apoptosis and senescence are important types of cell death following exposure to radiation that may be involved in the infiltration of macrophages to irradiated tissues [31]. Melatonin has shown the ability to attenuate radiation-induced apoptosis and senescence [32]. Preventing macrophage infiltration by melatonin, as observed in the current study, may be involved in the suppression of IL-4 and also other downstream genes including *IL4ra1*, *Duox1*, and *Duox2*. 

In recent years, some studies have been conducted to alleviate radiation toxicity in heart tissue. Amifostine, a food and drug administration (FDA) approved radioprotector, has shown some protective effects in rat’s heart tissues. However, it protects heart tissues only against some adverse effects, but not all of them [33]. In addition, some clinical studies have shown that treatment with amifostine may lead to severe toxicity in patients, hence a discontinuation of treatment [34]. Therefore, treatment with natural radioprotectors may be more effective due to their low toxicities and minimal side effects. In a previous study, we showed that hesperidin as a natural antioxidant has the ability to reduce radiation-induced heart injury. We also showed that the most obvious effect of hesperidin is related to preventing infiltration of macrophages in rat’s heart tissues [35]. In this study, we report for the first time the ability of melatonin to inhibit *Duox1* and *Duox2* in rat’s heart tissues. This may be proposed as a mechanism for radioprotection by melatonin.

## 5. Conclusions

This study has shown that irradiation of the hearts of rats leads to activation of IL-4 and its downstream genes: *IL4ra1*, *Duox1*, and *Duox2*. In addition, the expression of *Duox1* was increased without upregulation of IL-13, which may indicate a role of other mediators such as IL-4 in the expression of Duox1. Treatment with melatonin before irradiation could attenuate upregulation of IL-4, *IL4ra1*, *Duox1*, and *Duox2*. It also reversed the infiltration of inflammatory cells, including macrophages and lymphocytes. It is possible that melatonin via suppression of IL-4 and pro-oxidant enzymes including *Duox1* and *Duox2* protects against ionizing radiation-induced heart injury. As the release of IL-4 is highly dependent on the activity of macrophages, there is a possibility that melatonin reduces chronic inflammation via preventing macrophage infiltration and oxidative stress. As infiltration of macrophages is highly dependent on the induction of apoptosis and senescence following exposure to radiation, pre-irradiation administration of melatonin may reduce chronic infiltration of macrophages via the reduction of cell death.

## Figures and Tables

**Table 1:** Forward and reverse sequences of primers.

Gene	Forward Sequence	Reverse Sequence
*IL-4r1*	GAGTGAGTGGAGTCCCAGCATC	GCTGAAGTAACAGGTCAGGC
*Duox1*	AAGAAAGGAAGCATCAACACCC	ACCAGGGCAGTCAGGAAGAT
*Duox2*	AGTCTCATTCCTCACCCGGA	GTAACACACACGATGTGGCG
*PGM1*	CATGATTCTGGGCAAGCACG	GCCAGTTGGGGTCTCATACAAA

## References

[1] Eldabaje R., Le D.L., Huang W., Yang L.X. (2015). Radiation-associated Cardiac Injury. Anticancer Res..

[2] Kamiya K., Ozasa K., Akiba S., Niwa O., Kodama K., Takamura N., Zaharieva E.K., Kimura Y., Wakeford R. (2015). Long-term effects of radiation exposure on health. Lancet.

[3] Douple E.B., Mabuchi K., Cullings H.M., Preston D.L., Kodama K., Shimizu Y., Fujiwara S., Shore R.E. (2011). Long-term radiation-related health effects in a unique human population: Lessons learned from the atomic bomb survivors of Hiroshima and Nagasaki. Disaster Med. Public Health Prep..

[4] Sardaro A., Petruzzelli M.F., D’Errico M.P., Grimaldi L., Pili G., Portaluri M. (2012). Radiation-induced cardiac damage in early left breast cancer patients: Risk factors, biological mechanisms, radiobiology, and dosimetric constraints. Radiother. Oncol..

[5] Ghobadi G., van der Veen S., Bartelds B., de Boer R.A., Dickinson M.G., de Jong J.R., Faber H., Niemantsverdriet M., Brandenburg S., Berger R.M. (2012). Physiological interaction of heart and lung in thoracic irradiation. Int. J. Radiat. Oncol. Biol. Phys..

[6] Spetz J., Moslehi J., Sarosiek K. (2018). Radiation-induced cardiovascular toxicity: mechanisms, prevention, and treatment. Curr. Treat. Options Cardiovasc. Med..

[7] Boerma M., Wang J., Wondergem J., Joseph J., Qiu X., Kennedy R.H., Hauer-Jensen M. (2005). Influence of mast cells on structural and functional manifestations of radiation-induced heart disease. Cancer Res..

[8] Robbins M.E., Zhao W. (2004). Chronic oxidative stress and radiation-induced late normal tissue injury: A review. Int. J. Radiat. Biol..

[9] Seddon M., Looi Y.H., Shah A.M. (2007). Oxidative stress and redox signalling in cardiac hypertrophy and heart failure. Heart.

[10] Ameziane-El-Hassani R., Talbot M., Dos Santos M.C.D.S., Al Ghuzlan A., Hartl D., Bidart J.M., De Deken X., Miot F., Diallo I., de Vathaire F. (2015). NADPH oxidase DUOX1 promotes long-term persistence of oxidative stress after an exposure to irradiation. Proc. Natl. Acad. Sci. USA.

[11] Di Maggio F.M., Minafra L., Forte G.I., Cammarata F.P., Lio D., Messa C., Gilardi M.C., Bravatà V. (2015). Portrait of inflammatory response to ionizing radiation treatment. J. Inflamm..

[12] Carvalho D.P., Dupuy C. (2013). Role of the NADPH oxidases DUOX and NOX4 in thyroid oxidative stress. Eur. Thyr. J..

[13] Amini P., Kolivand S., Saffar H., Rezapoor S., Motevaseli E., Najafi M., Nouruzi F., Shabeeb D., Musa A.E. (2018). Protective effect of Selenium-L-methionine on radiation-induced acute pneumonitis and lung fibrosis in rat. Curr. Clin. Pharmacol..

[14] Monceau V., Pasinetti N., Schupp C., Pouzoulet F., Opolon P., Vozenin M.-C. (2010). Modulation of the Rho/ROCK pathway in heart and lung after thorax irradiation reveals targets to improve normal tissue toxicity. Curr. Drug Targets.

[15] Musa A.E., Shabeeb D. (2019). Radiation-induced heart diseases: protective effects of natural products. Medicina.

[16] Mihandoost E., Shirazi A., Mahdavi S.R., Aliasgharzadeh A. (2014). Can melatonin help us in radiation oncology treatments?. BioMed Res. Int..

[17] Ataee R., Shokrzadeh M., Jafari-Sabet M., Nasrabadi Nasri N., Ataie A., Haghi Aminjan H. (2017). The role of melatonin and melatonin receptors in pharmacology and pharmacotherapy of cancer. Austin Oncol..

[18] Najafi M., Shirazi A., Motevaseli E., Geraily G., Norouzi F., Heidari M., Rezapoor S. (2017). The melatonin immunomodulatory actions in radiotherapy. Biophys. Rev..

[19] Farhood B., Goradel N.H., Mortezaee K., Khanlarkhani N., Salehi E., Nashtaei M.S., Mirtavoos-Mahyari H., Motevaseli E., Shabeeb D., Musa A.E. (2019). Melatonin as an adjuvant in radiotherapy for radioprotection and radiosensitization. Clin. Transl. Oncol..

[20] Bedini A., Fraternale A., Crinelli R., Mari M., Bartolucci S., Chiarantini L., Spadoni G. (2019). Design, synthesis, and biological activity of hydrogen peroxide responsive arylboronate melatonin hybrids. Chem. Res. Toxicol..

[21] Conlon P.J., Tyler S., Grabstein K.H., Morrissey P. (1989). Interleukin-4 (B-cell stimulatory factor-1) augments the in vivo generation of cytotoxic cells in immunosuppressed animals. Biotechnol. Ther..

[22] Kioi M., Husain S.R., Croteau D., Kunwar S., Puri R.K. (2006). Convection-enhanced delivery of interleukin-13 receptor-directed cytotoxin for malignant glioma therapy. Technol. Cancer Res. Treat..

[23] Liang L., Hu D., Liu W., Williams J.P., Okunieff P., Ding I. (2003). Celecoxib reduces skin damage after radiation: Selective reduction of chemokine and receptor mRNA expression in irradiated skin but not in irradiated mammary tumor. Am. J. Clin. Oncol..

[24] Dadrich M., Nicolay N.H., Flechsig P., Bickelhaupt S., Hoeltgen L., Roeder F., Hauser K., Tietz A., Jenne J., Lopez R. (2016). Combined inhibition of TGFβ and PDGF signaling attenuates radiation-induced pulmonary fibrosis. Oncoimmunology.

[25] Groves A.M., Johnston C.J., Misra R.S., Williams J.P., Finkelstein J.N. (2016). Effects of IL-4 on pulmonary fibrosis and the accumulation and phenotype of macrophage subpopulations following thoracic irradiation. Int. J. Radiat. Biol..

[26] Esposito E., Cuzzocrea S. (2010). Antiinflammatory activity of melatonin in central nervous system. Curr. Neuropharmacol..

[27] Najafi M., Shirazi A., Motevaseli E., Geraily G., Amini P., Tooli L.F., Shabeeb D. (2019). Melatonin modulates regulation of NOX2 and NOX4 following irradiation in the lung. Curr. Clin. Pharmacol..

[28] Fernandez-Gil B., Moneim A.E., Ortiz F., Shen Y.Q., Soto-Mercado V., Mendivil-Perez M., Guerra-Librero A., Acuna-Castroviejo D., Molina-Navarro M.M., Garcia-Verdugo J.M. (2017). Melatonin protects rats from radiotherapy-induced small intestine toxicity. PLoS ONE.

[29] Chen Y., Zhao Q., Sun Y., Jin Y., Zhang J., Wu J. (2018). Melatonin induces anti-inflammatory effects via endoplasmic reticulum stress in RAW264.7 macrophages. Mol. Med. Rep..

[30] Meziani L., Deutsch E., Mondini M. (2018). Macrophages in radiation injury: A new therapeutic target. Oncoimmunology.

[31] Oishi Y., Manabe I. (2016). Macrophages in age-related chronic inflammatory diseases. NPJ Aging Mech. Dis..

[32] Zhou L., Chen X., Liu T., Gong Y., Chen S., Pan G., Cui W., Luo Z.P., Pei M., Yang H. (2015). Melatonin reverses H2 O2 -induced premature senescence in mesenchymal stem cells via the SIRT1-dependent pathway. J. Pineal Res..

[33] Gurses I., Ozeren M., Serin M., Yucel N., Erkal H.S. (2018). Histopathological efficiency of amifostine in radiation induced heart disease in rats. Bratisl. Lek Listy.

[34] Thorstad W.L., Chao K.S., Haughey B. (2004). Toxicity and compliance of subcutaneous amifostine in patients undergoing postoperative intensity-modulated radiation therapy for head and neck cancer. Semin. Oncol..

[35] Rezaeyan A., Haddadi G.H., Hosseinzadeh M., Moradi M., Najafi M. (2016). Radioprotective effects of hesperidin on oxidative damages and histopathological changes induced by X-irradiation in rats heart tissue. J. Med. Phys..

